# Two lymphoid cell lines potently silence unintegrated HIV-1 DNAs

**DOI:** 10.1186/s12977-022-00602-7

**Published:** 2022-07-09

**Authors:** Franziska K. Geis, Demetra P. Kelenis, Stephen P. Goff

**Affiliations:** 1 Department of Biochemistry and Molecular Biophysics, Columbia University Medical Center, New York, NY USA; 2 Department of Microbiology and Immunology, Columbia University Medical Center, New York, NY USA; 3 Howard Hughes Medical Institute, Columbia University Medical Center, New York, NY USA

**Keywords:** Unintegrated HIV-1 DNAs, Transcriptional silencing, Histone deposition and posttranslational histone modifications, Antiviral mechanisms, Lymphocytes

## Abstract

Mammalian cells mount a variety of defense mechanisms against invading viruses to prevent or reduce infection. One such defense is the transcriptional silencing of incoming viral DNA, including the silencing of unintegrated retroviral DNA in most cells. Here, we report that the lymphoid cell lines K562 and Jurkat cells reveal a dramatically higher efficiency of silencing of viral expression from unintegrated HIV-1 DNAs as compared to HeLa cells. We found K562 cells in particular to exhibit an extreme silencing phenotype. Infection of K562 cells with a non-integrating viral vector encoding a green fluorescent protein reporter resulted in a striking decrease in the number of fluorescence-positive cells and in their mean fluorescence intensity as compared to integration-competent controls, even though the levels of viral DNA in the nucleus were equal or in the case of 2-LTR circles even higher. The silencing in K562 cells was functionally distinctive. Histones loaded on unintegrated HIV-1 DNA in K562 cells revealed high levels of the silencing mark H3K9 trimethylation and low levels of the active mark H3 acetylation, as detected in HeLa cells. But infection of K562 cells resulted in low H3K27 trimethylation levels on unintegrated viral DNA as compared to higher levels in HeLa cells, corresponding to low H3K27 trimethylation levels of silent host globin genes in K562 cells as compared to HeLa cells. Most surprisingly, treatment with the HDAC inhibitor trichostatin A, which led to a highly efficient relief of silencing in HeLa cells, only weakly relieved silencing in K562 cells. In summary, we found that the capacity for silencing viral DNAs differs between cell lines in its extent, and likely in its mechanism.

**Supplementary Information:**

The online version contains supplementary material available at 10.1186/s12977-022-00602-7.

## Introduction

The retroviral life cycle can be divided into two phases: an early phase, which includes cellular entry, reverse transcription of the viral RNA into viral DNA and the stable integration into the target cell genome [1], and a late phase, which includes the expression of viral genomic RNA, mRNAs and viral protein precursors, the assembly and release of new viral progeny, and the final maturation step into a fully infectious virion [2]. This study focuses on the early steps of the retroviral life cycle occurring after nuclear entry of the viral DNA and before viral integration into the host chromosomes. During this time there exist three forms of unintegrated retroviral DNA: linear DNAs, which are the template for subsequent stable integration, and two circularized forms with either one copy of the long terminal repeats (1-LTR circles) or two tandem copies of the repeats (2-LTR circles) [3, 4]. It is known that prevention of stable integration leads to an accumulation of circularized forms [5–8]. Unintegrated viral DNAs are not able to replicate and therefore gradually vanish during host cell proliferation. Most cells suppress early viral transcripts from the unintegrated DNAs and efficient viral expression is only established after integration in permissive cells. Our lab and others have previously described and characterized the silencing of unintegrated viral DNAs of the murine leukemia virus (MLV) [9, 10] as well as of the human immunodeficiency virus type 1 (HIV-1) [11–13]. Unintegrated HIV-1 DNAs are rapidly loaded with core as well as linker histones upon nuclear entry and post-translational histone modifications are deposited very soon thereafter [11, 14]. The histone profile of unintegrated HIV-1 DNAs includes high levels of the silencing mark H3K9 trimethylation (H3K9me3) and low levels of H3 acetylation, an active gene marker [11]. Importantly, we previously found that the identical silencing machinery is not universally active against all extrachromosomal DNAs, since silencing factors acting on unintegrated MLV DNA did not target unintegrated HIV-1 [10, 13], and host factors acting on unintegrated HIV-1 DNA did not affect transcription of unintegrated MLV DNA [15]. Furthermore, some viruses encode proteins that suppress the silencing. The herpes virus ICP0, the hepatitis B virus HBx, the HTLV-1 Tax, and the HIV-1 Vpr all have the ability to enhance expression of incoming viral DNA [13, 16–19]. In the case of HIV-1, the Vpr protein has activity to overcome the silencing of virus expression from unintegrated DNAs in a wide range of cell types, likely by altering chromatin structure [13, 20–22]. In this study, we report that the uninhibited silencing of viral expression of unintegrated HIV-1 DNAs is not only virus-specific, but also differs quantitatively and qualitatively between cell types.

## Results

### Expression of unintegrated HIV-1 DNAs is almost completely silenced in lymphoid K562 and T-lymphocytic Jurkat cells

**Fig. 1:**
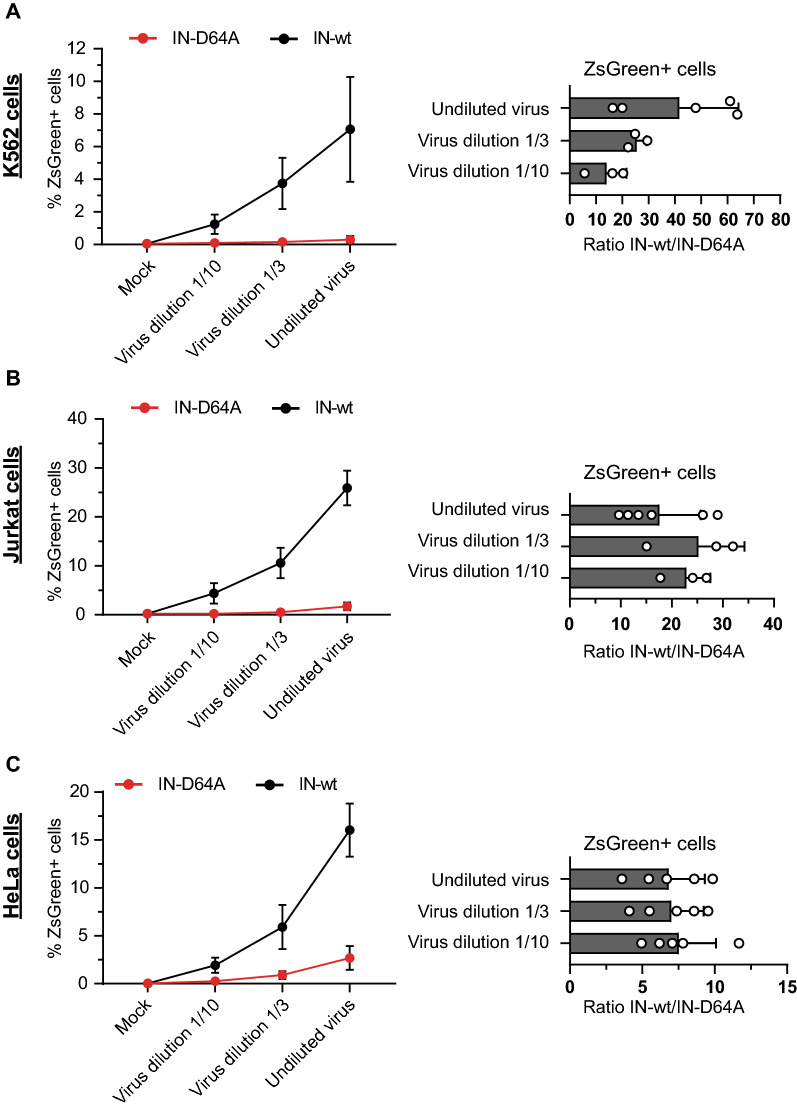
Lymphoid K562 and T-lymphocytic Jurkat cells profoundly silence unintegrated HIV-1 DNA. Cells were infected with different virus dilutions of IN-D64A or IN-wt virus as indicated. K562 cells (**A**), Jurkat cells (**B**) and HeLa cells (**C**) were harvested 24 h after virus application. The number of fluorescence-positive cells determined by flow cytometry (right panel) and the ratio of IN-wt and IN-D64A of the number of fluorescence-positive cells are shown (left panel). Error bars indicate mean ± SD. Mock represents uninfected control. Each dot represents an independent replicate

**Fig. 2:**
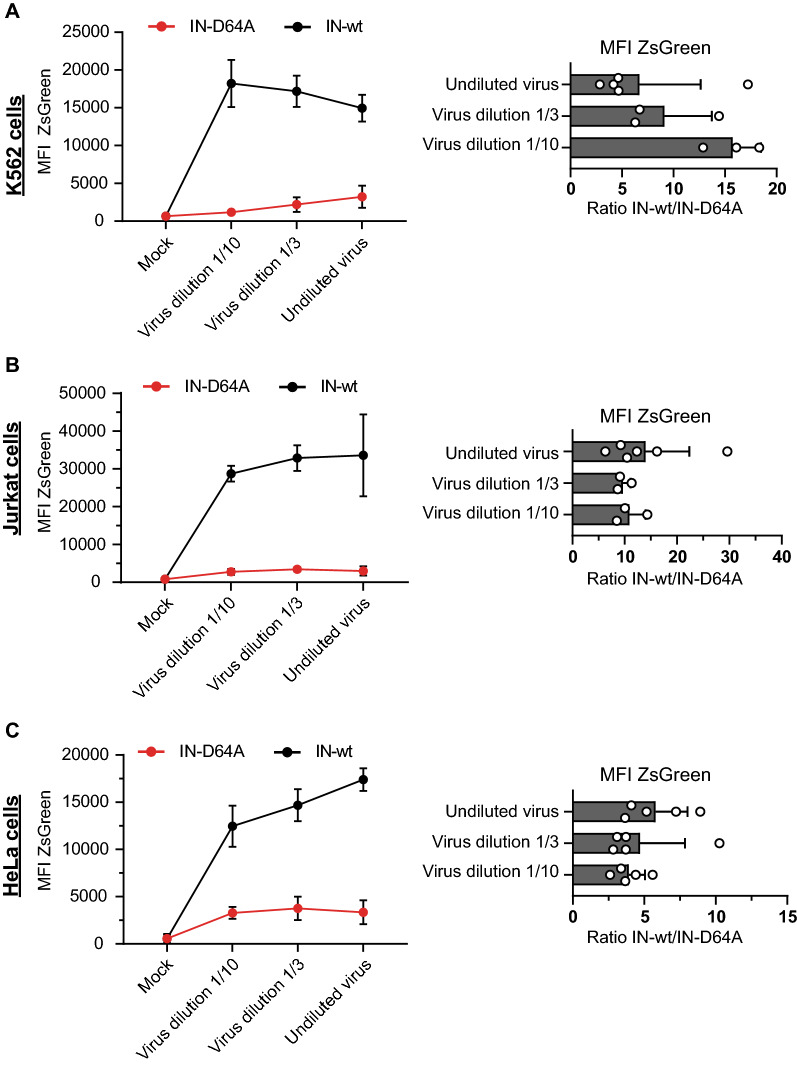
The mean fluorescence intensity (MFI) of fluorescence-positive cells is not a major part of the strong silencing phenotype. Cells were infected with different virus dilutions of IN-D64A or IN-wt virus as indicated. K562 cells (**A**), Jurkat cells (**B**) and HeLa cells (**C**) were harvested and the MFI of fluorescence-positive cells measured by flow cytometry 24 h after virus application (left panel). The ratio of IN-wt and IN-D64A of the MFI of fluorescence-positive cells is shown (right panel). Error bars indicate mean ± SD. Mock represents uninfected control. Each dot represents an independent replicate

In this study, we assayed expression of unintegrated viral DNAs using viral reporter constructs based on the pNL4-3.R^−^.E^−^ HIV-1 strain pseudotyped with the VSV-G envelope glycoprotein (VSV-G). We chose a viral genome lacking the Vpr gene to eliminate its functions in blocking the normal host silencing activities that we wished to monitor. Virus preparations were harvested from 293 T cells transfected with DNAs expressing the reporter genes and VSV-G, and then used to infect various target cells. To assure readouts only of expression of unintegrated HIV-1 DNA, we used a viral construct containing a point mutation in the viral integrase active site that prevents integration (IN-D64A). For comparative studies we used the equivalent virus preparations with the wild-type integrase (IN-wt). Both constructs contained a ZsGreen fluorescence cassette to allow the measurement of viral expression. Cells were infected with virus at various dilutions and scored for ZsGreen expression by flow cytometry at 24 h post infection. All tested cell types showed significantly suppressed expression of IN-D64A compared to IN-wt virus (Fig. 1A–C). The silencing was manifest in all cell types as a decreased number in ZsGreen-expressing cells as well as a decreased mean fluorescence intensity (MFI) of ZsGreen-expressing cells (Fig. 2A–C), consistent with previous studies [11–13]. But importantly, we found striking differences in the efficiency of silencing unintegrated HIV-1 DNAs between cells of lymphoid origin – K562 cells or T-lymphocytic Jurkat cells – and endothelial HeLa cells. As previously seen, HeLa cells showed substantial silencing, with the number of ZsGreen-positive cells of IN-D64A being approximately sevenfold lower than IN-wt across the applied virus dilutions (undiluted virus, virus dilution 1/3, virus dilution 1/10) (Fig. 1C). But the selective silencing of unintegrated DNA was dramatically higher in K562 (~ 27-fold) and Jurkat cells (~ 23-fold) (Fig. 1A + B). Indeed, while infection of K562 and Jurkat cells with IN-wt gave high levels of ZsGreen-positive cells, infection with IN-D64A virus showed very few ZsGreen-positive cells at all tested virus dilutions (Fig. 1B + C). The MFI of ZsGreen after infection by IN-D64A relative to IN-wt was reduced in all cell types, but reduced by a slightly larger factor in K562 and Jurkat cells (both ~ 11-fold) compared to HeLa cells (~ fivefold) (Fig. 2A–C).

**Fig. 3:**
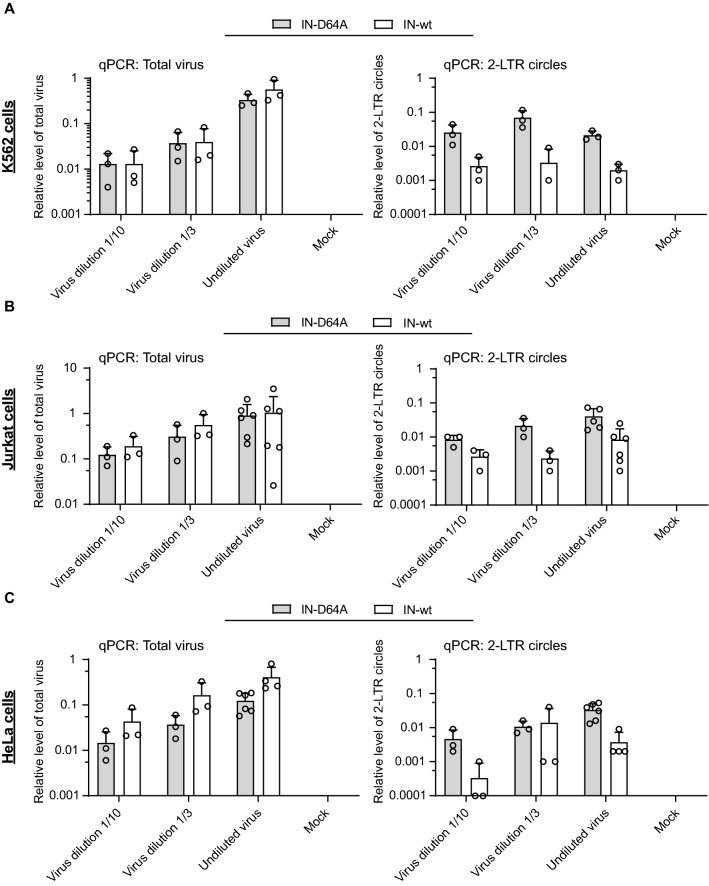
Total virus DNA levels after infection with IN-D64A and IN-wt virus are comparable in all cell lines. DNA was isolated from K562 cells (**A**), Jurkat cells (**B**) and HeLa cells (**C**) and the total viral DNA levels were determined by qPCR using ZsGreen-specific primers (left panel). 2-LTR circle levels were measured utilizing 2-LTR-specific primers (right panel). Levels are shown relative to GAPDH. Error bars indicate mean ± SD. Mock represents uninfected control. Each dot represents an independent replicate. All experiments were performed a minimum three times, and in many cases six times as indicated by the dots. The cases with no detected signal in the PCR runs lie below the X-axis in log scale data presentations (e.g. the Mock samples) and are left blank

To monitor the synthesis and the structures of the viral DNAs formed in the infected cells, we extracted DNA at 24 h after infection and carried out quantitative PCR (qPCR) reactions with primers specific for total viral DNA and 2-LTR circles. The levels of total viral DNA of IN-D64A and IN-wt were comparable in all cell types and at all tested virus doses (Fig. 3A–C, left panel). These data give assurance that the early steps of cellular entry and reverse transcription occurred normally, and that the lack of expression was not due to a lack of viral DNA. We detected 2-LTR circles with both constructs in all cell types, which indicates nuclear entry (Fig. 3A–C, right panel). The 2-LTR circle levels were increased in infections by IN-D64A compared to IN-wt in all cell types, but especially so in K562 cells (Fig. 3A–C, right panel). K562 gave the lowest viral expression from IN-D64A infection of all tested cell types despite having the highest levels of 2-LTR DNAs.

### Silencing of unintegrated HIV-1 DNAs is substantially relieved by the histone deacetylase (HDAC) inhibitor Trichostatin A (TSA) in HeLa cells but not in K562 cells

**Fig. 4:**
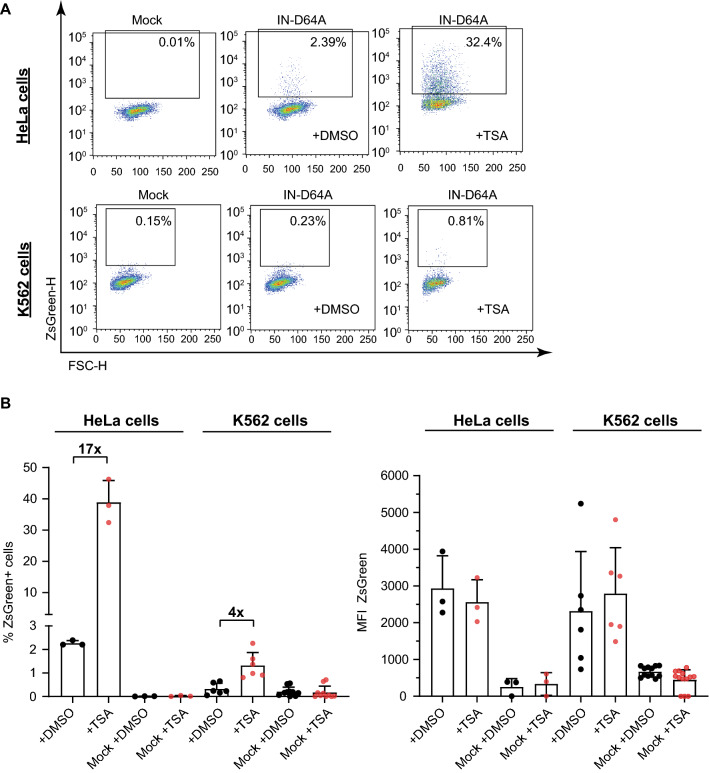
The histone deacetylase inhibitor TSA potently relieves silencing of unintegrated HIV-1 DNAs in HeLa cells but only weakly in lymphoid K562 cells. Cells were infected with IN-D64A in the presence of TSA or DMSO. Flow cytometry analyses were performed 24 h after virus application. **A** Representative flow cytometry plots are shown of infected HeLa and K562 cells treated with either TSA or DMSO. **B** Number of ZsGreen-positive cells (left panel) and the MFI of ZsGreen-positive cells are shown (right panel). Error bars indicate mean ± SD. Mock represents uninfected control. Each dot represents an independent replicate

The silencing of unintegrated viral DNAs in most settings is known to be reversed by the HDAC inhibitor TSA [23, 24]. The extreme level of silencing of unintegrated HIV-1 DNAs in K562 cells raised the possibility that distinctive mechanisms of action were involved, and that the silencing might not be responsive to TSA. To test this possibility, we treated K562 and HeLa cells with TSA and monitored viral expression 24 h after infection. Surprisingly, we observed substantial differences between these two cell types. Whereas the silencing of IN-D64A was potently relieved in HeLa cells upon TSA treatment (number of fluorescence-positive cells increased by up to 17-fold) (Fig. 4), viral expression in K562 cells was only increased by about fourfold and the number of ZsGreen-positive cells still remained very low at 2 percent or less (Fig. 4A, B left panel). These data suggest that the silencing machinery of unintegrated HIV-1 DNAs and the extent of silencing efficiency differ between cell types. We note that TSA had only an effect on the number of ZsGreen-expressing cells, but had no influence on the MFI of ZsGreen in both HeLa as well as K562 cells (Fig. 4A, B right panel).

### Histone profile of K562 cells is dominated by high silencing mark H3K9me3 and low active H3 acetylation

**Fig. 5:**
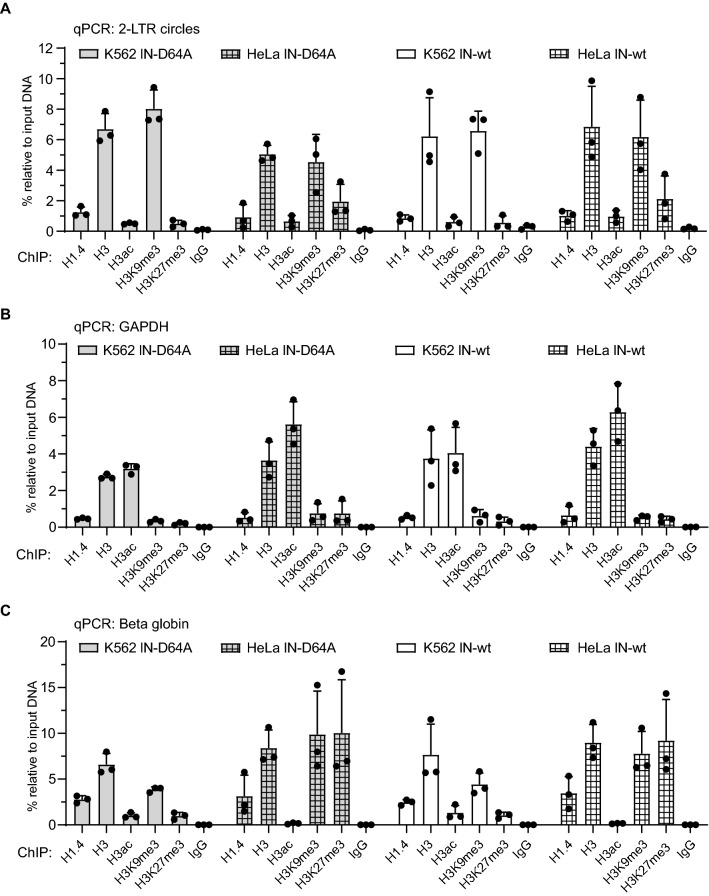
Histone profile of viral DNA in lymphoid K562 cells resembles that of transcriptionally inactive genes. K562 cells and HeLa cells were infected with IN-D64A or IN-wt reporter virus. Cells were harvested for ChIP 24 h after virus application. Antibodies against specific histones or histone modifications were used. Rabbit IgG was used as an isotype control. ChIP samples were analyzed by qPCR using 2-LTR-specific primers (**A**), GAPDH-specific primers (**B**) or beta globin-specific primers (**C**). Data are shown relative to input DNA. Error bars indicate mean ± SD. Each dot represents an independent replicate

To obtain a histone profile of unintegrated HIV-1 DNAs in K562 cells, we conducted chromatin immunoprecipitation assays (ChIP) and subsequent qPCR 24 h after infection with IN-D64A and IN-wt virus. HeLa cells served as a control cell type. Additionally, GAPDH and beta globin genes were used as active and inactive gene controls. We utilized antibodies specific for a range of histones, histone isotypes, and histone modifications. We detected H1.4 linker as well as H3 core histones on unintegrated HIV-1 DNA in K562 and HeLa cells (Fig. 5A). A notable difference of histone modifications between K562 and HeLa cells was the level of the silencing mark H3K27me3. Whereas H3K27me3 was detected on unintegrated viral DNA in HeLa cells, the amount of H3K27me3 in K562 cells remained low (Fig. 5A). The amount of the heterochromatin control beta globin DNA marked with the histone modification H3K27me3, however, was also low in K562 cells, suggesting a lower abundance of the mark in K562 cells in general (Fig. 5C). Thus, it is not clear whether the altered levels of this silencing mark is specific only to incoming viral DNAs or a general feature of chromosomal DNA of this cell line.

## Discussion

In this study, we analyzed and compared viral expression of unintegrated HIV-1 DNAs early in infection in various cell types. We found that K562 and Jurkat cells exhibited a markedly higher capacity for silencing incoming HIV-1 DNAs than HeLa cells. K562 cells in particular displayed an extremely effective histone-based transcriptional silencing phenotype: viral expression of integration-deficient virus was almost non-existent, despite an increased amount of 2-LTR circular DNAs. It was of interest that K562 and Jurkat cells are of lymphoid origin. While it has been a longstanding observation that most cell types suppress retroviral expression before integration [25, 26], there might be particularly strong selective pressure for the natural target cells of HIV-1 to avoid early viral expression as soon as possible.

The significance of the silencing of viral gene expression by the host is highlighted by the fact that HIV-1, like many viruses, has acquired mechanisms to suppress the silencing. The viral Vpr protein, brought into the cell within the virion particle, potently inactivates the silencing machinery and restores high-level expression [20–22]. We have compared Vpr-plus and Vpr-minus reporter genomes in HeLa cells and confirm the ability of Vpr to restore expression of integrase-deficient genomes in our assays (see Additional file 1: Fig. S1). The importance of unintegrated DNA forms in infected patients, and the origin and stability of these DNAs, are uncertain. Some investigators suggest that the DNAs are unstable and that their levels reflect very recent ongoing infections, while others suggest they can be stable for long periods; in either case they may act as templates for limited persistent transcriptional activity. This expression of unintegrated DNA, likely dependent on Vpr, may be distinctive for primary CD4 T cells and macrophages [6, 7, 27–29], supporting our indications of cell-type variability.

Another significant finding of this study is that the specific characteristics normally associated with silencing of unintegrated retroviral DNAs differed among the cell types. The pronounced silencing phenotype in K562 cells is not relieved as fully by TSA inhibition of deacetylation as in HeLa cells. In addition, the silencing marks placed on the histones of the viral DNA are not identical. The findings are consistent with recent findings that distinctive host factors are required for silencing in different cell types [13, 15]. These observations expand on earlier findings that the silencing machinery active on unintegrated MLV DNAs is not active in silencing of HIV-1 DNAs [10, 13]. Thus the silencing of invading viruses is distinctive both across cell types and substantially different between viruses.

Epigenetic and epitranscriptomic regulatory pathways which specifically target viral transcripts have the potential to play important roles for antiviral drug therapy [30]. Better understanding of the silencing of incoming DNAs may also help to optimize transient gene delivery options based on retroviruses, where low efficiencies of expression in relevant cell types such as stem or blood cells are still major hurdles to overcome in gene therapy approaches.

## Material and methods

### Retroviral reporter plasmids

HIV-1-based reporter virus encoded on plasmid pNL4-3.ZsGreen.R^−^.E^−^ was used throughout the study [11]. The integration-deficient version is here named as IN-D64A, and the wild-type integration-proficient version as IN-wt. Viral particles were pseudotyped with the vesicular stomatitis virus glycoprotein (VSV-G), encoded by pMD2.G (a gift from Didier Trono, École Polytechnique Fédérale de Lausanne, Lausanne, Switzerland; Addgene plasmid, 12259).

### Cells and cultivation

Human HeLa cells (American Type Culture Collection; #CCL-2) and human Lenti-X 293 T cells (Clontech; #632,180) were cultured in Dulbecco’s Modified Eagle Media (DMEM), supplemented with 10% heat-inactivated fetal bovine serum, 100 U/ml penicillin, and 100 µg/ml streptomycin. K562 cells (#CCL-243; American Type Culture Collection) were cultured in Iscove’s Modified Dulbecco’s Medium (IMDM) (#12–726; Lonza), supplemented with 10% heat-inactivated fetal bovine serum, 100 U/ml penicillin, 100 µg/ml streptomycin and 2 mM L-glutamine. Jurkat cells (Clone E6-1 #TIB-152; American Type Culture Collection) were cultured in Roswell Park Memorial Institute (RPMI) 1640 Medium (#22,400–105; Life Technologies), supplemented with 10% heat-inactivated fetal bovine serum, 100 U/ml penicillin, 100 µg/ml streptomycin and 2 mM L-glutamine.

### Retroviral particle production and infection

Virus production was performed in 293 T cells by calcium phosphate precipitation-based transfection method as previously described [11]. Viral supernatants were 100 × concentered by ultracentrifugation (2 h, 25,000 rpm, 4 °C) and stored at -80 °C. Virus production of integration-deficient and integration-proficient reporter viruses were done side-by-side. 5 × 10^4^ HeLa cells were seeded one day before infection, 7 × 10^4^ K562 or Jurkat cells were seeded at the day of infection, all in a 12-well format with 0.5 ml per well. Equal amounts of viral supernatants were used to infect cells for comparative studies, at the indicated range of dilutions (virus dilution 1/3 or 1/10 or undiluted virus). Virus was removed 5 h after virus application and replaced with fresh medium.

### Flow cytometry

Percentage of ZsGreen-positive cells and mean fluorescence intensities were measured with BD LSRII flow cytometer (BD Biosciences). Data were analyzed with FlowJo software (BD Biosciences). Cells were gated for viable cells with FSC-H/SSC-H and FSC-H/FITC-H was used to gate ZsGreen-positive population. MFI was determined after gating for the ZsGreen-positive population.

### qPCR

Cells were harvested 24 h after infection and DNA was isolated with QIAamp DNA blood mini kit (Qiagen) according manufacturer’s instructions. DNA concentrations were adjusted to 50 ng/µl and approximately 50–100 ng DNA per sample was used for qPCR analyses based on SYBR Green (Roche, #4913850001). Following primer pairs were used for 2-LTR circle detection (for 5’ AACTAGGGAACCCACTGCTTAAG 3’, rev 5’ TCCACAGATCAAGGATATCTTGTC 3’), ZsGreen-specifc primers as a readout for total viral DNA (for 5’ CCCCGTGATGAAGAAGATGA 3’, rev 5’ GTCAGCTTGTGCTGGATGAA 3’) and GAPDH primers as a housekeeping control (for 5’ CAATTCCCCATCTCAGTCGT 3 ‘, rev 5’ TAGTAGCCGGGCCCTACTTT 3 ‘). Quantitative PCRs were performed with 7500 Fast Real-Time PCR System (Applied Biosystems) (50 °C for 2 min, 95 °C for 10 min, 40 cycles of 15 s at 95 °C, 30 s at 60 °C and 30 s at 72 °C). A melting curve for quality control was generated for every run following manufacturer’s advice. The Ct-values were normalized to endogenous GAPDH levels using the 2^ΔCt^ method.

### ChIP

2 × 10^6^ HeLa cells and 4 × 10^6^ K562 cells were seeded one day before infection. Virus supernatants (15 µl) were pre-treated with 5 U/ml DNase I (#M6101; Promega) for 1 h at 37 °C. Medium was supplemented with 8 µg/ml Polybrene (#TR-1003-G; Millipore-Sigma). Virus and Polybrene were removed after 5 h. Cells were washed twice and harvested for ChIP approximately 24 h after infection. ChIP protocol was performed as previously described [11]. Following ChIP-grade antibodies were used with a concentration of 5 µg per 50 µg sonicated chromatin: Anti-H1.4 ([D4J5Q]; #41328S; Cell Signaling Technology); Anti-H3 (#ab1791; Abcam); Anti-H3K9me3 (#ab8898; Abcam); Anti H3 acetyl K9 + K14 + K18 + K23 + K27 (#ab47915; Abcam); Anti-H3K27me3 (#ab192985; Abcam); Rabbit IgG isotype control (#02-6102; Invitrogen). 5 µl of eluted DNA per sample were used for qPCR. PCR protocol and primers were used as described above. Additionally, beta globin specific primers served as a heterochromatin control (for 5’ CAGAGCCATCTATTGCTTAC 3’, rev: 5’ GCCTCACCACCAACTTCATC 3’). Data were determined relative to input DNA with the 2^ΔCt^ method and presented as percentage relative to input DNA in the bar graphs.

## Supplementary Information

**Additional file 1: Figure S1.** Viral protein Vpr relieves silencing of unintegrated HIV-1 DNA. HeLa cells were infected with HIV-1 reporter virus expressing ZsGreen, either integration-competent (IN-wt, black curves) or integration-deficient (IN-D64A, red curves). A Vpr-positive reporter genome was generated by restoring the ORF sequence to the parent viral genome. Cells were scored by flow cytometry for % ZsGreen (upper panels) and Mean Fluorescence Intensity (MFI, lower panels). **A** Vpr-minus vector. **B** Vpr-positive vector.

## Data Availability

The datasets used and/or analysed during the current study are available from the corresponding author on reasonable request.

## Abbreviations

LTR: Long Terminal Repeat
H3K27: Histone H3 lysine 27
H3K9me3: Histone H3 lysine 9 trimethylation
HIV-1: Human immunodeficiency virus type 1
HDAC: Histone deacetylase
TSA: Trichostatin A
MLV: Murine leukemia virus
VSV-G: Vesicular stomatitis virus glycoprotein
