# Supplementary material for: Two lymphoid cell lines potently silence unintegrated HIV-1 DNAs
Source: Retrovirology. 2022 Jul 9;19:16. doi: 10.1186/s12977-022-00602-7 (PMC9271240; doi:10.1186/s12977-022-00602-7)
Supplement: Supplementary file 1 — Additional file 1: Figure S1. Viral protein Vpr relieves silencing of unintegrated HIV-1 DNA. HeLa cells were infected with HIV-1 reporter virus expressing ZsGreen, either integration-competent (IN-wt, black curves) or integration-deficient (IN-D64A, red curves). A Vpr-positive reporter genome was generated by restoring the ORF sequence to the parent viral genome. Cells were scored by flow cytometry for % ZsGreen (upper panels) and Mean Fluorescence Intensity (MFI, lower panels). A Vpr-minus vector. B Vpr-positive vector. [file 12977_2022_602_MOESM1_ESM.zip › 12977_2022_602_MOESM1_ESM/Legend Figure S1.pdf]

Supplementary Figure S1

Viral protein Vpr relieves silencing of unintegrated HIV-1 DNA. HeLa cells were infected with HIV-1 viral vectors expressing ZsGreen, and either integration-competent (IN-wt, black curves) or integration-deficient (IN-D64A, red curves). Cells were scored by flow cytometry for % ZsGreen (upper panels) and Mean Fluorescence Intensity (lower panels). Panels A: Vectors were Vpr-minus. Panels B: Vectors were Vpr-plus.
